# Evaluation of an early step-down strategy from intravenous anidulafungin to oral azole therapy for the treatment of candidemia and other forms of invasive candidiasis: results from an open-label trial

**DOI:** 10.1186/1471-2334-14-97

**Published:** 2014-02-21

**Authors:** Jose Vazquez, Annette C Reboli, Peter G Pappas, Thomas F Patterson, John Reinhardt, Peter Chin-Hong, Ellis Tobin, Daniel H Kett, Pinaki Biswas, Robert Swanson

**Affiliations:** 1 Georgia Regents University, Augusta, GA, USA; 2 Cooper Medical School of Rowan University, Camden, NJ, USA; 3 University of Alabama at Birmingham, Birmingham, AL, USA; 4 University of Texas and South Texas Veterans Health Care System, San Antonio, TX, USA; 5 Christiana Care Health Services, Newark, DE, USA; 6 University of California San Francisco, San Francisco, CA, USA; 7 Albany Medical Center, Albany, NY, USA; 8 University of Miami Miller School of Medicine and Jackson Memorial Hospital, Miami, FL, USA; 9 Pfizer Inc, New York, NY, USA

**Keywords:** Anidulafungin, Azole, Candidemia, Step-down strategy

## Abstract

**Background:**

Hospitalized patients are at increased risk for candidemia and invasive candidiasis (C/IC). Improved therapeutic regimens with enhanced clinical and pharmacoeconomic outcomes utilizing existing antifungal agents are still needed.

**Methods:**

An open-label, non-comparative study evaluated an intravenous (IV) to oral step-down strategy. Patients with C/IC were treated with IV anidulafungin and after 5 days of IV therapy had the option to step-down to oral azole therapy (fluconazole or voriconazole) if they met prespecified criteria. The primary endpoint was the global response rate (clinical + microbiological) at end of treatment (EOT) in the modified intent-to-treat (MITT) population (at least one dose of anidulafungin plus positive *Candida* within 96 hours of study entry). Secondary endpoints included efficacy at other time points and in predefined patient subpopulations. Patients who stepped down early (≤ 7 days’ anidulafungin) were identified as the "early switch" subpopulation.

**Results:**

In total, 282 patients were enrolled, of whom 250 were included in the MITT population. The MITT global response rate at EOT was 83.7% (95% confidence interval, 78.7–88.8). Global response rates at all time points were generally similar in the early switch subpopulation compared with the MITT population. Global response rates were also similar across multiple *Candida* species, including *C. albicans*, *C. glabrata*, and *C. parapsilosis*. The most common treatment-related adverse events were nausea and vomiting (four patients each).

**Conclusions:**

A short course of IV anidulafungin, followed by early step-down to oral azole therapy, is an effective and well-tolerated approach for the treatment of C/IC.

**Trial registration:**

ClinicalTrials.gov: NCT00496197

## Background

Candidemia and invasive candidiasis (C/IC) are important causes of morbidity and complications in patient populations including neutropenic patients, solid-organ and stem-cell transplant recipients, patients with indwelling intravascular devices, and patients in intensive care. The incidence of candidemia appears to be considerably higher in the United States (US) compared with Canada and Europe, with the exception of Denmark [1,2]. The attributable mortality of candidemia in US studies has ranged from 19% to 49% [3,4]. Over the past decade, the incidence of candidal infections due to non-*albicans Candida* (NAC) species that are resistant to fluconazole has increased, highlighting the need for optimized antifungal regimens.

An initial short course of an intravenous (IV) echinocandin, followed by the option to step-down to oral azole therapy, could be as effective for the treatment of C/IC as conventional 10- to 14-day IV regimens, including for the rarer *Candida* species. Additionally, this step-down strategy could have added benefits, such as better tolerability, reduced use of IV catheters, earlier patient discharge, and significant cost savings, as documented in oral step-down antibiotic regimens [5-11], although prospective data for de-escalation in antifungal therapy are lacking.

In vitro, the echinocandins (including anidulafungin) have reported potent fungicidal activity against most *Candida* species and demonstrated a favorable safety profile [12-18]. Echinocandins are recommended as the treatment of choice for severely ill and neutropenic patients with proven or suspected invasive candidiasis in recent clinical practice guidelines [13]. An azole, such as fluconazole, is widely used and recommended as a first-line agent for the treatment of C/IC in non-critically ill patients [13]. Voriconazole is also recommended as a step-down therapy for patients with infection due to some of the NAC species following first-line treatment with an echinocandin or amphotericin B.

In patients with candidemia, continual therapy until 14 days after the first negative blood culture is recommended. The Infectious Diseases Society of America (IDSA) guidelines include the recommendation to step-down to oral azole therapy as early as possible once the patient is clinically stable and blood cultures have become negative [13]. However, this specific strategy is based on limited clinical evidence for antifungal treatment and has not been prospectively studied. The European Society for Clinical Microbiology and Infectious Diseases (ESCMID) suggests simplification of treatment by stepping down to oral fluconazole after 10 days of treatment if the patient is stable, and tolerates oral therapy, and if the *Candida* species is susceptible [19]. However neither of these specific strategies have been prospectively studied and the appropriate timing of step-down therapy remains unclear.

Recognizing this gap in current knowledge, we evaluated the efficacy and safety of utilizing initial therapy with IV anidulafungin, followed by an optional early step-down to oral fluconazole or voriconazole for the treatment of C/IC in adults. The study was specifically designed to evaluate anidulafungin treatment in a broad group of patients with C/IC caused by various species of *Candida* and to explore use of an early step-down strategy in a real-world setting.

## Methods

### Study design

This was a Phase IV, open-label, noncomparative study, involving 44 centers across the US and four centers in the Republic of Korea. The four Korean centers were added to increase enrollment and because treatment practices for invasive candidiasis were similar to those in the US. The trial was conducted in accordance with the ethical principles decreed by the Declaration of Helsinki and in compliance with International Conference on Harmonization Good Clinical Practice Guidelines. The final protocol, any amendments, and the informed consent document were reviewed and approved by the Institutional Review Boards and/or Independent Ethics Committees at each of the investigational centers (Additional file 1: Table S1). All patients or their proxy provided written informed consent prior to study initiation.

Patients ≥ 18 years of age were enrolled in the study if the presence of candidemia (positive blood culture) or invasive candidiasis (positive culture from a normally sterile site) was demonstrated from a culture obtained within 96 hours of study entry. In addition, the presence of one or more of the following signs and symptoms of infection was also required: fever or hypothermia; hypotension; localized signs and symptoms of inflammation; or radiological findings of invasive candidiasis. Patients were allowed to participate if they had received no more than 48 hours of systemic azole therapy. Prior prophylaxis with azoles was permitted provided it was discontinued prior to the study. Patients were excluded if they had one of the following: life expectancy of less than 1 month; hypersensitivity to echinocandins or azoles; prior treatment with either an echinocandin or amphotericin B; systemic antifungal therapy had failed for the current episode of C/IC; a diagnosis of fungal endophthalmitis with involvement of the vitreous humor; chronic refractory neutropenia and were not expected to recover (defined as absolute neutrophil count < 500 cells/mm^3^ for 28 days prior to the baseline visit); or the presence of confirmed or suspected *Candida* osteomyelitis, endocarditis, or meningitis. Removal of IV catheters suspected to be the cause of infection was required within 24 hours of starting study drug. To allow for delays in scheduling catheter removal in these seriously ill patients, patients who had all catheters removed or replaced to another anatomical location by study Day 3 were considered to have had their catheters removed.

### Study treatment

All patients received 200 mg IV anidulafungin as a single loading dose and 100 mg IV anidulafungin daily thereafter for a maximum of 28 days. After 5 days’ IV anidulafungin, investigators could transition patients to an oral azole if they met the following criteria: ability to tolerate oral therapy; afebrile for > 24 hours; hemodynamically stable; not neutropenic; and had documented clearance of *Candida* from the bloodstream. Patients were not randomized or pre-selected to receive early oral step down therapy; rather the decision was made based on the patient’s condition and protocol guidelines. Per protocol, patients with positive baseline cultures for *C. albicans* and *C. parapsilosis* were stepped down to oral fluconazole (400 mg/day); all other patients were stepped down to oral voriconazole (200 mg twice daily). Combined IV and oral agents were given for at least 14 days after the last positive blood/tissue culture.

### Study assessments

#### Patient populations

The following efficacy analysis sets were considered:

*Intent-to-treat (ITT) population*, all subjects who had taken at least one dose of study drug. All safety analyses were based on the ITT population.

*Modified intent-to-treat (MITT) population*, all ITT subjects with a positive baseline culture for a *Candida* species. Missing and unknown global responses were not included in the analysis. Patients in the MITT population were further categorized into those who stepped down to oral azole therapy and those who remained on IV therapy.

*Early switch subpopulation*, patients who stepped down to oral azole therapy by Day 7 of the study (Days 1 and 2 of potential switch to oral therapy). Missing and unknown global responses were not included in the analysis.

An additional *sensitivity analysis* was performed, which set missing values and unknown responses as failures. This allowed a more direct comparison between studies, as the global response rates assessed in previous anidulafungin studies set missing and unknown values to failure [17].

#### Efficacy

Blood cultures were performed at the screening visit and repeated daily until Day 5, and at the end of treatment (EOT) visit. If a positive culture was obtained on Day 5, a repeat culture was required on Day 7. Patients with candidemia whose blood culture remained positive on Day 7 were considered treatment failures and were discontinued from the study. Susceptibility testing was conducted on all baseline isolates using the Clinical Laboratory Standards Institute M27 microbroth dilution method and M27-A3 breakpoints.

The primary efficacy endpoint was the global response rate (clinical + microbiological response) at EOT based on the MITT population. A successful global response was defined as both clinical success (*cure* – resolution of signs and symptoms of *Candida* infection, or *improvement* – significant, but incomplete, resolution of signs and symptoms of *Candida* infection) and microbiological success (*eradication* – negative follow-up culture for *Candida* species, or *presumed eradication* – follow-up culture was not available and clinical response was defined as cure or improvement). Global responses were also assessed independently by a data adjudication committee (DAC), which periodically reviewed certain specified endpoints as defined separately in a charter, but the assessments by the investigator were considered the primary endpoint.

In the primary MITT analysis, global response rates at EOT and end of study (EOS) were calculated with missing/unknown outcomes excluded. However, sensitivity analyses were also performed, in which missing/unknown outcomes were set to failure.

Key secondary endpoints and other parameters of the study included: global response rate at secondary time points, such as the end of IV (EOIV) treatment, at 2 weeks post-treatment, and at EOS (6 weeks post-therapy); time to negative blood culture; global response rate for the subset of subjects with NAC infections; and all-cause and attributable mortality.

#### Safety

Clinical and laboratory evaluations were also performed periodically throughout the study. Routine safety assessments were made during IV and oral treatment as well as during the follow-up period. All adverse events (AEs) and serious adverse events (SAEs) were monitored.

#### Post-hoc analyses

Baseline characteristics, global response rates at EOT, and secondary time points for the early switch subpopulation were assessed alongside all MITT patients.

#### Statistical analyses

The planned sample size was 286 patients, which, assuming an evaluability rate of 70% and a 50:50 distribution of *albicans* to NAC infections, would result in approximately 100 patients with baseline infections caused by non-*albicans* species. There were no formal pre-specified statistical decision rules, as this was a single-arm estimation study. No formal hypotheses were tested in this study and the design did not allow for statistical comparisons to be made between the early switch subpopulation and the MITT population.

## Results

### Patient disposition

**Figure 1:**
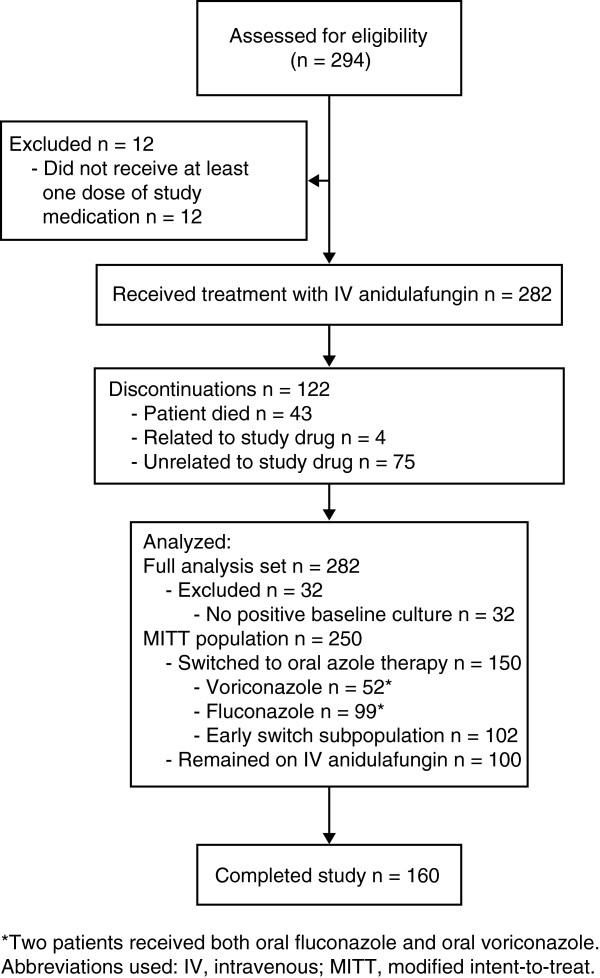
CONSORT diagram.

In total, 294 patients were screened for entry into the study (270 in US, 24 in Korea), and 282 patients were enrolled and received treatment. The study was conducted between July 2007 and June 2010. Patient disposition is shown in Figure 1. In the MITT population, 54% were male, with a mean (standard deviation [SD]) age of 55.4 (16.9) years, and mean (SD) Acute Physiology and Chronic Health Evaluation (APACHE)-II score of 14.3 (6.7). *C. albicans* (45%) was the most common baseline pathogen isolated. For the early switch subpopulation (step-down within 7 days of starting treatment with anidulafungin), demographics and characteristics were generally similar to the MITT population.

The APACHE-II score was lower in the early switch subpopulation (mean [SD] 12.7 [6.2]) compared with the MITT population. Patient demographics and baseline characteristics for both the MITT population and the early switch subpopulation are summarized in Table 1.

**Table 1:** Combined summary of demography and baseline characteristics of the MITT population and early switch subpopulation

	**MITT population**	**Early switch subpopulation**
**Patient demographics**		
Number of patients	250	102
Female (%)	115 (46.0)	49 (48.0)
Mean age (SD)	55.4 (16.9)	53.9 (17.7)
Median age (range)	56.0 (19–89)	53.0 (19–88)
Race (%)		
White	164 (65.6)	69 (67.6)
Black	52 (20.8)	28 (27.5)
Asian	26 (10.4)	1 (1.0)
Other	8 (3.2)	4 (3.9)
**Baseline characteristics**		
Number of patients (%)	250 (100.0)	102 (100.0)
Baseline site of infection (%)		
Blood only	210 (84.0)	86 (84.3)
Other sterile site	38 (15.2)	16 (15.7)
Blood and other sterile site	2 (0.8)	0
APACHE-II score		
n (%)	249 (99.6)	102 (100.0)
Mean (SD)	14.3 (6.7)	12.7 (6.2)
Median (range)	14.0 (2–36)	12.0 (2–29)
> 20 (%)	47 (18.8)	14 (13.7)
ANC		
n (%)	227 (90.8)	93 (91.2)
≤ 500 cells/mm^3^ (%)	9 (3.6)	2 (2.0)
Length of ICU stay ≥ 4 days (%)*	82 (34.3)	23 (23.5)
Invasive candidiasis related to IV catheter*		
Yes (%)	104 (45.8)	44 (46.3)
All catheters removed within 3 days of therapy?		
Yes	98 (94.2)	41 (93.2)
No	6 (5.8)	3 (6.8)
No (%)	41 (18.1)	14 (14.7)
All catheters removed within 3 days of therapy?		
Yes	25 (61.0)	8 (57.1)
No	16 (39.0)	6 (42.9)
**Baseline isolates of *Candida *** **species, *n *** **(%)**		
*C. albicans*	112 (44.8)	46 (45.1)
*C. glabrata*	64 (25.6)	20 (19.6)
*C. parapsilosis*	41 (16.4)	20 (19.6)
*C. tropicalis*	27 (10.8)	11 (10.8)
*C. krusei*	11 (4.4)	4 (3.9)
Other	9 (3.6)	6 (5.9)
**Most commonly reported comorbidities (terms reported from ≥ 10%), n (%)**		
Anemia	127 (50.8)	58 (56.9)
Nausea	70 (28.0)	29 (28.4)
Diabetes mellitus	48 (19.2)	27 (26.5)
Hypokalemia	51 (20.4)	26 (25.5)
Malnutrition	49 (19.6)	24 (23.5)

*Risk factors for invasive candidiasis and catheter management data were systematically collected only after Case Report Form (CRF) revision shortly after study start. Percentages are based on the actual number of patients enrolled in the study after these CRFs were incorporated.

*Abbreviations used*: ANC, absolute neutrophil count; APACHE, Acute Physiology and Chronic Health Evaluation; ICU, intensive care unit; IV, intravenous; MITT, modified intent-to-treat; SD, standard deviation.

Of the 250 MITT patients, 100 (40%) remained on anidulafungin treatment throughout the study and had a median duration of 12.0 days of therapy (range, 1–29). One hundred and fifty (60%) of the MITT patients stepped down to oral azole therapy, with a median duration of 6.0 days of parenteral therapy (Table 2). There were 102 patients in the early switch subpopulation; the median duration of IV therapy prior to oral step-down was 5.0 days (range, 1–6) and the median duration of overall antifungal therapy was 14.0 days (range, 5–56). There were 48 patients in the late switch subpopulation; the median duration of IV therapy prior to oral step-down was 10.0 days (range, 6–27) and the median duration of overall antifungal therapy was 19.0 days (range, 8–42). The durations of therapy for the MITT population, and the early switch subpopulation, late switch subpopulation, and no switch subpopulation are shown in Table 2.

**Table 2:** Duration of therapy (MITT, early switch subpopulation, late switch subpopulation, and no switch subpopulation)

	**MITT population**			**Early switch subpopulation**			**Late switch subpopulation**			**No switch subpopulation**		
	**(n = 250)**			**(n = 102)**			**(n = 48)**			**(n = 100)**		
	**n (%)**	**Median, days**	**Range, days**	**n (%)**	**Median, days**	**Range, days**	**n (%)**	**Median, days**	**Range, days**	**n (%)**	**Median, days**	**Range, days**
Duration of overall therapy (IV + oral)	250 (100)	14.0	1-56	102 (100)	14.0	5-56	48 (100)	19.0	8-42	100 (100)	12.0	1-29
Duration of IV anidulafungin portion of therapy*	250 (100)	6.0	1-29	102 (100)	5.0*	1-6*	48 (100)	10.0	6-27	100 (100)	12.0	1-29
Study day of step-down to oral therapy	150 (60)	6.0	1-28	102 (100)	6.0	1-7	48 (100)	11.0	8-28	-	-	-
1-5	7 (4.7)	-	-	7 (6.9)	-	-	-	-	-	-	-	-
6	77 (51.3)	-	-	77 (75.5)	-	-	-	-	-	-	-	-
7	18 (12.0)	-	-	18 (17.6)	-	-	-	-	-	-	-	-
8-14	38 (25.3)	-	-	-	-	-	38 (79.2)	-	-	-	-	-
15-21	6 (4.0)	-	-	-	-	-	6 (12.5)	-	-	-	-	-
22-28	4 (2.7)	-	-	-	-	-	4 (8.3)	-	-	-	-	-

*Represents the duration of IV therapy prior to oral step-down. Four patients from the early switch subpopulation had to be stepped back to IV therapy after initially stepping down to oral azole as they later became unable to tolerate oral medication. Total duration of IV therapy for these patients is, therefore, longer than reported here.

*Abbreviations used*: IV, intravenous; MITT, modified intent-to-treat.

### Efficacy

The global response rates at EOT were as follows: 83.7% (95% confidence interval [CI], 78.7–88.8; 170/203 patients) in the MITT; 89.9% (95% CI, 86.0–93.8) as assessed by the DAC; and 68.0% (95% CI, 62.2–73.8; 170/250 patients) for the sensitivity analysis. The global response rates for the primary analysis and sensitivity analyses at EOIV, 2 weeks post-treatment, and EOS are shown in Table 3.

**Table 3:** Responses at EOT and secondary timepoints in the MITT population and early switch subpopulation

	**MITT population**	**Early switch subpopulation**
	**(n = 250)**	**(n = 102)**
**Response**	**n/N (%) [95% CI]**^**a**^	**n/N (%) [95% CI]**^**a**^
Global response at EOT		
Success	170/203 (83.7) [78.7–88.8]	81/ 90 (90.0) [83.8–96.2]
Sensitivity analysis*	170/250 (68.0) [62.2–73.8]	81/102 (79.4) [71.6–87.3]
Failure	33	9
Missing/unknown	47	12
Clinical response at EOT		
Success	174/187 (93.0) [89.4–96.7]	83/ 89 (93.3) [88.0–98.5]
Sensitivity analysis*	174/250 (69.6) [63.9–75.3]	83/102 (81.4) [73.8–88.9]
Failure	13	6
Missing/unknown	63	13
Microbiological response at EOT		
Success	183/192 (95.3) [92.3–98.3]	87/ 90 (96.7) [93.0–100.0]
Sensitivity analysis*	183/250 (73.2) [67.7–78.7]	87/102 (85.3) [78.4–92.2]
Failure	9	3
Missing/unknown	58	12
Global response at secondary time points	**n/N (%) [95% CI]**	**n/N (%) [95% CI]**
EOIV	208/235 (88.5) [84.4–92.6]	97/101 (96.0) [92.2–99.8]
Sensitivity analysis*	208/250 (83.2) [78.6–87.8]	97/102 (95.1) [90.9–99.3]
Week 2 follow-up	148/194 (76.3) [70.3–82.3]	72/ 86 (83.7) [75.9–91.5]
Sensitivity analysis*	148/250 (59.2) [53.1–65.3]	72/102 (70.6) [61.7–79.4]
EOS	131/187 (70.1) [63.5–76.6]	68/ 86 (79.1) [70.5–87.7]
Sensitivity analysis*	131/250 (52.4) [46.2–58.6]	68/102 (66.7) [57.5–75.8]

*Missing/unknown values set as failures; ^a^95% CI based on normal approximation to the binomial.

*Abbreviations used*: CI, confidence interval; EOIV, end of intravenous treatment; EOS, end of study; EOT, end of treatment; MITT, modified intent-to-treat.

In the early switch subpopulation (≤ 7 days’ anidulafungin), the global response rate at EOT was 90.0% (95% CI, 83.8–96.2) and 79.4% (95% CI, 71.6–87.3) for the sensitivity analysis. In general, patients in the early switch subpopulation had global response rates that were higher than the MITT population at all time points (Table 3).

The highest successful global response rates at EOT were observed in patients with *C. albicans* (87.0% [95% CI, 80.1–93.8]) and *C. glabrata* (86.5% [95% CI, 77.3–95.8]) infections, while the lowest response rates were observed in patients with *C. krusei* (60.0% [95% CI, 29.6–90.4]) isolated at baseline. Global response rates for the MITT and early switch subpopulations at EOT and secondary time points per baseline pathogen are shown in Table 4. In the early switch subpopulation there were no failures in patients with *C. tropicalis* or *C. krusei* infection throughout the study time points, although the numbers were small (9 and 3 patients at EOT for *C. tropicalis* or *C. krusei*, respectively).

**Table 4:** Global response rates by baseline pathogen in the MITT population and early switch subpopulation

	**MITT population**		**Early switch subpopulation**	
**Baseline pathogen**	**EOT**	**EOS**	**EOT**	**EOS**
	**n (%)**	**n (%)**	**n (%)**	**n (%)**
	[95% CI]*	[95% CI]*	[95% CI]*	[95% CI]*
*C. albicans*				
Subjects in analysis	92	84	41	40
Success	80 (87.0)	63 (75.0)	36 (87.8)	33 (82.5)
	[80.1–93.8]	[65.7–84.3]	[77.8–97.8]	[70.7–94.3]
Failure	12 (13.0)	21 (25.0)	5 (12.2)	7 (17.5)
*C. glabrata*				
Subjects in analysis	52	48	18	17
Success	45 (86.5)	31 (64.6)	16 (88.9)	10 (58.8)
	[77.3–95.8]	[51.1–78.1]	[74.4–100.0]	[35.4–82.2]
Failure	7 (13.5)	17 (35.4)	2 (11.1)	7 (41.2)
*C. parapsilosis*				
Subjects in analysis	34	32	18	18
Success	26 (76.5)	22 (68.8)	16 (88.9)	15 (83.3)
	[62.2–90.7]	[52.7–84.8]	[74.4–100.0]	[66.1–100.0]
Failure	8 (23.5)	10 (31.3)	2 (11.1)	3 (16.7)
*C. tropicalis*				
Subjects in analysis	19	17	9	8
Success	15 (78.9)	12 (70.6)	9 (100.0)	8 (100.0)
	[60.6–97.3]	[48.9–92.2]	[100.0–100.0]	[100.0–100.0]
Failure	4 (21.1)	5 (29.4)	0	0
*C. krusei*				
Subjects in analysis	10	10	3	3
Success	6 (60.0)	5 (50.0)	3 (100.0)	3 (100.0)
	[29.6–90.4]	[19.0–81.0]	[100.0–100.0]	[100.0–100.0]
Failure	4 (40.0)	5 (50.0)	0	0

*95% CI based on normal approximation to the binomial.

*Abbreviations used*: CI, confidence interval; EOS, end of study; EOT, end of treatment; MITT, modified intent-to-treat.

Median time to negative blood culture for all patients was two days (with Day 1 being the first dose of study drug), and approximately 90% of patients achieved a negative blood culture by Day 5. All but six baseline isolates were susceptible to anidulafungin and only one of the patients with a resistant baseline pathogen was a treatment failure. Three patients receiving oral fluconazole and four patients receiving oral voriconazole had baseline isolates which were resistant to fluconazole and voriconazole, respectively. There were no treatment failures among these seven patients. Baseline isolates from all other patients were documented susceptible to the oral agents used.

### Safety

In total, 33 (11.7%) patients reported a total of 59 treatment-related AEs. Nausea and vomiting were the most frequently reported treatment-related AEs, both reported in four patients (1.4%). Of the 13 patients who permanently discontinued from the study, only one discontinuation was anidulafungin-related (an episode of vomiting). Three patients experienced SAEs related to treatment with anidulafungin as judged by the site principal investigator – acute renal failure, abnormal liver function test, and systemic *Candida.* One patient receiving voriconazole was identified with a treatment-related SAE case of *C. difficile* colitis, and one patient was identified with acute renal failure, hypotension, and dehydration as a treatment-related SAE with fluconazole.

There were 65 deaths (23.0%) recorded in the 282 subjects receiving at least one dose of anidulafungin. Forty-three of these deaths occurred during the study period (Figure 1). Twenty subjects died after withdrawing from the study for a variety of reasons and two subjects died after completing the study. None of the deaths were considered to be related to study drug or treatment regimens. There were 14 deaths (13.7%) recorded in the 102 subjects in the early switch population.

## Discussion

The early step-down from an echinocandin to an oral azole among stable patients with IC is a suggested strategy in the recently published treatment guidelines for C/IC [13]. However, this recommendation is based on limited clinical evidence, expert opinion, and common practice. Indeed, in recent trials of the treatment of candidemia, step-down to an azole was prohibited before 10 days of parenteral therapy with an echinocandin [14,17,20].

In this study, a step-down strategy from IV to oral therapy was incorporated as part of the study design. Sixty percent (60%) of patients enrolled in this study underwent early step-down (by Day 7) to either fluconazole or voriconazole. Although baseline characteristics for the early switch subpopulation were generally similar to the overall MITT population, some interesting differences were noted. In the early switch subpopulation compared with the overall MITT population, *C. glabrata* isolates were less common. Importantly, fewer early switch patients had an APACHE-II score >20 and fewer had a length of intensive care unit stay ≥4 days. This suggests that patients in the early switch subpopulation were less severely ill. In general, the early switch subpopulation showed response rates similar to the MITT population and these response rates were maintained through the end of study. These results demonstrate the efficacy of including an early step-down strategy in treating patients with C/IC.

Direct comparison across trials should be regarded cautiously; however, global response rates in both the MITT population and the early step-down subpopulation from this study were comparable to other trials that evaluated anidulafungin for the treatment of C/IC [14-17]. This included the study by Reboli and colleagues, which demonstrated higher response rates for anidulafungin compared to fluconazole for the treatment of C/IC [17]. The most relevant parameter in comparing efficacy results with those in the Reboli et al. study was the global response in the sensitivity analysis (missing/unknown set to failure). In the current study, the global response rates in the sensitivity analysis at EOT for the MITT and early step-down subpopulation were 68% and 79.4%, respectively. This compared with a global response rate at EOIV of 75.6% from the Reboli et al. study. Furthermore, the survival rates and safety profile in the current study were comparable to the Reboli et al. study and other previously reported clinical trials [14-17]. This comparison suggests that a C/IC treatment regimen utilizing anidulafungin in a strategy of early step-down yields similar efficacy and outcome results when compared to previous studies that required a minimum 10 days of parenteral anidulafungin therapy.

The early step-down approach used in the current study was effective against a wide range of *Candida* species. Successful response rates were comparable to the rates seen in *C. albicans* infections in patients with either *C. glabrata* or *C. parapsilosis* infections, the second and third most common pathogens isolated in this patient population. Additionally, failures were not reported for *C. tropicalis* or *C. krusei* infections in the early switch subpopulation. Successful global response rates across all time points were similar for voriconazole and fluconazole (data not shown), indicating that either drug could be employed as a step-down therapy after IV anidulafungin.

One hundred patients with candidemia remained on IV anidulafungin throughout the study. These patients tended to be more severely ill at baseline, as reflected in a higher APACHE-II score, or were more likely to have had recent abdominal surgery and the concern about adequacy of oral absorption.

The high global response rate (88.5%) at EOIV is likely to be reflective of the high level of activity of initial treatment with anidulafungin; this is further supported by the rapid eradication of *Candida* from blood cultures. In contrast to prior candidemia studies, daily blood cultures were required (Days 1–5), allowing for a rigorous assessment of culture eradication times. The median time to blood culture eradication in this study was two days, consistent with the in vitro fungicidal activity of anidulafungin and other echinocandins against most *Candida* species [21].

Based on the findings of this study, patients with C/IC are suitable candidates for step-down oral antifungal therapy provided the *Candida* has been cleared from the bloodstream, the patient is clinically stable and capable of taking oral therapy. The advantages of the approach, beyond its convenience and flexibility, include possible additional clinical benefits, such as shorter duration or need for intravascular catheters, decreased length of stay and a potential cost saving, as documented for other, antibiotic, step-down regimens [5-11].

Limitations of the study include the fact that the study was not appropriately designed for statistical comparison between the early switch subpopulation and the MITT. Likewise, there was no controlling for baseline severity factors between these two populations. Additionally, this was an open-label study, therefore there was no active or placebo comparator for the response rates observed.

## Conclusions

A strategy utilizing initial therapy with anidulafungin for 5 days followed by an optional step-down to oral fluconazole or voriconazole, based on isolated *Candida* species, is an effective and well-tolerated regimen and could be employed as an alternative treatment regimen for patients with C/IC. Shorter transition times may be possible dependent upon earlier laboratory results and patient response rates to initial anidulafungin parenteral therapy. An IV to oral step-down strategy may have a favorable impact on removal of intravascular devices, hospital length of stay, and cost.

## Abbreviations

AE: Adverse event; ANC: Absolute neutrophil count; APACHE: Acute Physiology and Chronic Health Evaluation; C/IC: Candidemia and invasive candidiasis; CI: Confidence interval; CRF: Case Report Form; DAC: Data adjudication committee; EOIV: End of intravenous treatment; EOS: End of study; EOT: End of treatment; ICU: Intensive care unit; IV: Intravenous; MITT: Modified intent-to-treat; NAC: Non-*albicans Candida*; SAE: Serious adverse event; SD: Standard deviation.

## Competing interests

PB and RS are employees of Pfizer Inc. DHK is a consultant for Pfizer, and has received honoraria or speaking fees from Pfizer Inc, Astellas, and GlaxoSmithKline, and grants from Pfizer Inc and Akers Bioscience. PGP has acted as an advisor for and has received research grant support from Pfizer Inc, Merck, Astellas, Gilead, and T2 Biosystems. ACR has received research grant support and acted as a lecturer and consultant for Pfizer Inc. JV has received research grant support from Pfizer Inc, Merck, and Astellas. TP has served as a consultant for Pfizer Inc, Merck, Astellas, Toyoma and Viamet, and has received research grants from Pfizer Inc, Merck and Astellas.

## Authors’ contributions

PB and RS conceived and designed the study. PB carried out the statistical analysis. PM, PRH, JV, AR, PP, TP, JR, PC-H, ET, DK performed the study. All authors reviewed and approved the final manuscript.

## Pre-publication history

The pre-publication history for this paper can be accessed here:

http://www.biomedcentral.com/1471-2334/14/97/prepub

## Supplementary Material

Additional file 1: Table S1 List of investigators and corresponding institutional review boards or independent ethics committees. Click here for file

## References

[B1] Pfaller MA Diekema DJ Epidemiology of invasive candidiasis: a persistent public health problem Clin Microbiol Rev 2007 14 133 163 10.1128/CMR.00029-06 17223626 PMC1797637

[B2] Vincent JL Rello J Marshall J Silva E Anzueto A Martin CD Moreno R Lipman J Gomersall C Sakr Y Reinhart K International study of the prevalence and outcomes of infection in intensive care units JAMA 2009 14 2323 2329 10.1001/jama.2009.1754 19952319

[B3] Gudlaugsson O Gillespie S Lee K Vande Berg J Hu J Messer S Herwaldt L Pfaller M Diekema D Attributable mortality of nosocomial candidemia, revisited Clin Infect Dis 2003 14 1172 1177 10.1086/378745 14557960

[B4] Morgan J Meltzer MI Plikaytis BD Sofair AN Huie-White S Wilcox S Harrison LH Seaberg EC Hajjeh RA Teutsch SM Excess mortality, hospital stay, and cost due to candidemia: a case–control study using data from population-based candidemia surveillance Infect Control Hosp Epidemiol 2005 14 540 547 10.1086/502581 16018429

[B5] Sevinc F Prins JM Koopmans RP Langendijk PN Bossuyt PM Dankert J Speelman P Early switch from intravenous to oral antibiotics: guidelines and implementation in a large teaching hospital J Antimicrob Chemother 1999 14 601 606 10.1093/jac/43.4.601 10350396

[B6] Drew RH Antimicrobial stewardship programs: how to start and steer a successful program J Manag Care Pharm 2009 14 S18 S23 19236137 10.18553/jmcp.2009.15.s2.18 PMC10437655

[B7] Chalmers JD Al-Khairalla M Short PM Fardon TC Winter JH Proposed changes to management of lower respiratory tract infections in response to the *Clostridium difficile* epidemic J Antimicrob Chemother 2010 14 608 618 10.1093/jac/dkq038 20179023

[B8] Mertz D Koller M Haller P Lampert ML Plagge H Hug B Koch G Battegay M Fluckiger U Bassetti S Outcomes of early switching from intravenous to oral antibiotics on medical wards J Antimicrob Chemother 2009 14 188 199 10.1093/jac/dkp131 19401304 PMC2692500

[B9] Nicolau DP Containing costs and containing bugs: are they mutually exclusive? J Manag Care Pharm 2009 14 S12 17 19236136 10.18553/jmcp.2009.15.s2.12 PMC10438171

[B10] Desai M Franklin BD Holmes AH Trust S Richards M Jacklin A Bamford KB A new approach to treatment of resistant gram-positive infections: potential impact of targeted IV to oral switch on length of stay BMC Infect Dis 2006 14 94 10.1186/1471-2334-6-94 16762061 PMC1513579

[B11] McLaughlin CM Bodasing N Boyter AC Fenelon C Fox JG Seaton RA Pharmacy-implemented guidelines on switching from intravenous to oral antibiotics: an intervention study QJM 2005 14 745 752 10.1093/qjmed/hci114 16126741

[B12] Denning DW Echinocandin antifungal drugs Lancet 2003 14 1142 1151 10.1016/S0140-6736(03)14472-8 14550704

[B13] Pappas PG Kauffman CA Andes D Benjamin DK Jr Calandra TF Edwards JE Jr Filler SG Fisher JF Kullberg BJ Ostrosky-Zeichner L Reboli AC Rex JH Walsh TJ Sobel JD Clinical practice guidelines for the management of candidiasis: 2009 update by the Infectious Diseases Society of America Clin Infect Dis 2009 14 503 535 10.1086/596757 19191635 PMC7294538

[B14] Krause DS Reinhardt J Vazquez JA Reboli A Goldstein BP Wible M Henkel T Phase 2, randomized, dose-ranging study evaluating the safety and efficacy of anidulafungin in invasive candidiasis and candidemia Antimicrob Agents Chemother 2004 14 2021 2024 10.1128/AAC.48.6.2021-2024.2004 15155194 PMC415613

[B15] Krause DS Simjee AE van Rensburg C Viljoen J Walsh TJ Goldstein BP Wible M Henkel T A randomized, double-blind trial of anidulafungin versus fluconazole for the treatment of esophageal candidiasis Clin Infect Dis 2004 14 770 775 10.1086/423378 15472806

[B16] Vazquez JA Schranz JA Clark K Goldstein BP Reboli A Fichtenbaum C A phase 2, open-label study of the safety and efficacy of intravenous anidulafungin as a treatment for azole-refractory mucosal candidiasis J Acquir Immune Defic Syndr 2008 14 304 309 10.1097/QAI.0b013e31817af47a 18545153

[B17] Reboli AC Rotstein C Pappas PG Chapman SW Kett DH Kumar D Betts R Wible M Goldstein BP Schranz J Krause DS Walsh TJ Anidulafungin versus fluconazole for invasive candidiasis N Engl J Med 2007 14 2472 2482 10.1056/NEJMoa066906 17568028

[B18] Kett DH Shorr AF Reboli AC Reisman AL Biswas P Schlamm HT Anidulafungin compared with fluconazole in severely ill patients with candidemia and other forms of invasive candidiasis: support for the 2009 IDSA treatment guidelines for candidiasis Crit Care 2011 14 R253 10.1186/cc10514 22026929 PMC3334804

[B19] Cornely OA Bassetti M Calandra T Garbino J Kullberg BJ Lortholary O Meersseman W Akova M Arendrup MC rikan-Akdagli S Bille J Castagnola E Cuenca-Estrella M Donnelly JP Groll AH Herbrecht R Hope WW Jensen HE Lass-Florl C Petrikkos G Richardson MD Roilides E Verweij PE Viscoli C Ullmann AJ ESCMID* guideline for the diagnosis and management of Candida diseases 2012: non-neutropenic adult patients Clin Microbiol Infect 2012 14 Suppl 7 19 37 23137135 10.1111/1469-0691.12039

[B20] Mora-Duarte J Betts R Rotstein C Colombo AL Thompson-Moya L Smietana J Lupinacci R Sable C Kartsonis N Perfect J Caspofungin Invasive Candidiasis Study Group Comparison of caspofungin and amphotericin B for invasive candidiasis N Engl J Med 2002 14 2020 2029 10.1056/NEJMoa021585 12490683

[B21] Pfaller MA Diekema DJ Ostrosky-Zeichner L Rex JH Alexander BD Andes D Brown SD Chaturvedi V Ghannoum MA Knapp CC Sheehan DJ Walsh TJ Correlation of MIC with outcome for *Candida* species tested against caspofungin, anidulafungin, and micafungin: analysis and proposal for interpretive MIC breakpoints J Clin Microbiol 2008 14 2620 2629 10.1128/JCM.00566-08 18579718 PMC2519503

