# Supplementary material for: Evaluation of an early step-down strategy from intravenous anidulafungin to oral azole therapy for the treatment of candidemia and other forms of invasive candidiasis: results from an open-label trial
Source: BMC Infect Dis. 2014 Feb 21;14:97. doi: 10.1186/1471-2334-14-97 (PMC3944438; doi:10.1186/1471-2334-14-97)
Supplement: Additional file 1: Table S1 — List of investigators and corresponding institutional review boards or independent ethics committees. [file 1471-2334-14-97-S1.doc]

# Additional file 1: Table S1. List of investigators and corresponding Institutional Review Boards or Independent Ethics Committees

| **Center** | **Principal investigator** | **Sub-investigator** | **Address** | **Institutional Review Board or Independent Ethics Committee address** |
| --- | --- | --- | --- | --- |
| 1053 | Doo Ryeon Chung | Young Eun Ha; Eun-Jeong Joo; Dr. Mi-Kyong Joung; Chul Won Jung; Jeong-A Lee; Prof. Nam Yong Lee; Soo-youn Moon; Prof. Kyong Ran Peck; Kyung Mok Sohn | Samsung Medical Center  Division of Infectious Diseases 50 Irwon-dong, Gangnam-gu Seoul, 135-710 Republic of Korea | Samsung Medical Center IRB  50 Irwon-dong, Gangnam-Gu Seoul, 135-710 Republic of Korea |
| 1054 | Yang Soo Kim | Dr. Mi-Na Kim; Sung-Han Kim;Ki-Ho Park; Eun Hee Song | Asan Medical Center Division of Infectious Diseases  388-1 Pungnap-2dong, Songpa- gu Seoul, 138-736 Republic of Korea | IRB of Asan Medical Center 388-1 Pungnap-2dong, Songpa-gu Seoul, 138-736 Republic of Korea |
| 1055 | Chang Oh Kim;  Dr. Jun Yong Choi (Previous PI) | Ji-hyeon Baek; Yun Tae Chae; Bum Sik Chin; Injoo Cho; Heekyoung Choi; Sang Hoon Han; Dr. Su Jin Jeong; Han-Sung Lee | Yonsei University College of Medicine Severance  Department of Internal Medicine 250 Seongsanno, Seodaemoon- Gu Seoul, 120-752 Republic of Korea | IRB of Severance Hospital Yonsei University College of Medicine Severance hospital 250 Seongsanno, Seodaemun-Gu Seoul, 120-752 Republic of Korea |
| 1060 | Prof. Dong-Gun Lee | Kwon Jae-Cheol; Dr. Si-Hyun Kim | The Catholic University of Korea Seoul St. Mary's Hospital Division of Infectious Disease 505 Banpo-dong, Seocho-gu Seoul, 137-701 Republic of Korea | IRB of The Catholic University of Korea Seoul St. Mary's Hospital  505 Banpo-dong, Seocho-gu Seoul, 137-701 Republic of Korea |
| 1001 | Dr. Jose A. Vazquez | Dr. Anne Y. Chen; Dr. Ameet Hingwe; Dr. Norman Peter Markowitz; Dr. Mayur Ramesh; Theresa Sheppard; Suzanne E. Woodrich | Henry Ford Hospital 2799 West Grand Blvd Detroit, MI 48202 United States | IRB of Henry Ford Hospital 2799 West Grand Boulevard Detroit, MI 48202 United States |
| 1002 | Dr. Byungse Suh | Dr. Ji Hoon Baang; Dr. Edward Joseph Blanchard; Dr. Jason J. Bofinger; Dr. Heather E. Clauss; Dr. Heather Lynne Nace; Dr. Angela Jasmine Oates; Dr. Jaclyn Aileen Rosenzweig | Temple University Hospital 3401 North Broad Street Philadelphia, PA 19140 United States | Western IRB Inc. 3535 7th Avenue, SW Olympia, WA 98502 United States |
| 1003 | Dr. Robert Frank Betts | Melissa M. Khorana; Dr. William H. Novak; Dr. Sumanth Rajagopal | University of Rochester Medical Center 601 Elmwood Avenue Rochester, NY 14642 United States | Western IRB Inc. 3535 7th Avenue, SW Olympia, WA 98502 United States |
| 1004 | Dr. Daniel Hugh Kett | Dr. Michele Ilene Morris; Dr. Andrew Alan Quartin; Dr. Roland Milo Schein | University of Miami-Jackson Memorial Hospital Central Building, 4th Floor Room C455D 1611 NW 12th Avenue Miami, FL 33136 United States | University of Miami Human Subjects Research Office Suite 1002 1500 NW 12th Avenue Miami, FL 33136 United States |
| 1005 | Dr. John R. Pullman | Dr. David P. Chamberlain;  Eric C. Leber; Erin L. Williams-Leber | Mercury Street Medical Group  300 West Mercury Street Butte, MT 59701 United States  St. James Healthcare  400 South Clark Street Butte, MT 59701 United States | Western IRB Inc. 3535 7th Avenue, SW Olympia, WA 98502 United States |
| 1006 | Dr. Robert S. Jones Jr | Dr. Wendy L. Babitt; Dr. Edward Joseph Blanchard; Brian D. Couch; Crystal L. Nagle; Dr. Debra Powell; Dr. Ann Kyungwohn Shin; Susan C. Swonger; Dr. Ambreen Umer | RPS Infectious Diseases Suite 130 301 South 7th Avenue West Reading, PA 19611 United States  The Reading Hospital and Medical  Center 6th Avenue & Spruce Street West Reading, PA 19611 United States | IRB of The Reading Hospital and Medical Center  6th Avenue & Spruce Street N-Ground West Reading, PA 19611 United States |
| 1007 * | Dr. Shirish Huprikar | Dr. Michael Lief | Mount Sinai Medical Cente /Mount Sinai School of Medicine  1 Gustave L. Levy Place New York, NY 10029 United States | IRB of Mount Sinai School of Medicine  Box 1075 1 Gustave Levy Place New York, NY 10029 United States |
| 1008 * | Dr. Bruce C. Friedman | Dr. Claus Brandigi; Dr. Zaheed Hassan; Dr. Robert F. Mullins; Dr. Joseph R. Shaver | Doctors Hospital Augusta 3651 Wheeler Road Augusta, GA 30909 United States  Joseph M. Still Research Foundation Inc. 4A George C. Wilson Court Augusta, GA 30909 United States | Western IRB Inc. 3535 7th Avenue, SW Olympia, WA 98502 United States |
| 1010 | Dr. Paul Peniston Cook | Dr. Titu D. Das; Dr. Carlos O. Guerra; Shradha Pokharel; Dr. Madhvi Rana; Dr. Paras C. Udani | Pitt County Memorial Hospital 2100 Stantonsburg Road Greenville, NC 27834 United States  The Brody School of Medicine at East Carolina Univeristy Division of Infectious Diseases Doctors Park 6A Greenville, NC 27834 United States | IRB of University & Medical Center  East Carolina University Ed Warren Life Sciences Building Room 104 Greenville, NC 27834 United States |
| 1011 * | Dr. Priscilla Bayani Sioson | Dr. Julie T. Antique; Dr. Debra Lee Rainey | Jackson-Madison County General Hospital 708 West Forest Avenue Jackson, TN 38301 United States  West Tennessee Transitional Care 670 Skyline Drive Jackson, TN 38301 United States | IRB of Jackson-Madison County General Hospital  708 West Forest Avenue Jackson, TN 38301 United States |
| 1013 | Dr. Annette C. Reboli | Dr. John Dutton Baxter; Dr. Sumit Bhutani; Dr. Pola de la Torre; Dr. Henry Shalom Fraimow; Dr. Rose Kim; Dr. Diane M. Marchesani; Dr. Daniel K. Meyer; Dr. Mir A. Mohammad; Dr. Jennifer Patterson; Dr. Rosalie Pepe; Mary Pileggi; Dr. Manish Rai; Dr. Divya Sareen; Dr. Amanda Sevrin; Dr. Ahmad L. Subhi; Dr. Jacqueline D. Sutton; Dr. Constantine Tsigrelis; | Cooper University Hospital Suite 513 3 Cooper Plaza Camden, NJ 08103 United States  Cooper University Hospital One Cooper Plaza Camden, NJ 08103 United States | IRB of Cooper University Hospital Suite 504 3 Cooper Plaza Camden, NJ 08103 United States |
| 1014 * | Dr. John R. Perfect | Dr. Barbara Dudley Alexander; Dr. J. Andrew Alspaugh; Dr. Richard H. Drew; Dr. Melissa D. Johnson; Dr. John C. Yang; Dr. Aimee Kirsch Zaas | Duke University Medical Center, Duke Hospital Clinics Trent Drive Durham, NC 27710 United States  Duke University Medical Center, Hospital North Erwin Road Durham, NC 27710 United States | IRB of Duke University Health System Box 2991 Durham, NC 27710 United States |
| 1015 | Dr. Ellis Harris Tobin | Dr. Philip J. Palmieri | Albany Medical Center Hospital 47 New Scotland Avenue Albany, NY 12208 United States | Western IRB Inc. P O Box 12029 3535 7th Avenue, SW Olympia, WA 98502 United States |
| 1016 | Dr. Howard Belzberg |  | LAC+USC Medical Center 1200 North State Street Los Angeles, CA 90033 United States | IRB of USC Health Sciences Suite 4700 1200 North State Street Los Angeles, CA 90033 United States |
| 1017 | Dr. Peter George Pappas | Dr. John William Baddley; Dr. Matthew J. McCall; Dr. Mukesh Patel | Infectious Disease Clinic 908 20th Street South Birmingham, AL 35294 United States  Kirklin Clinic 2000 6th Avenue South Birmingham, AL 35233 United States  University of Alabama at Birmingham 619 19th Street South Birmingham, AL 35249 United States | Western IRB Inc. 3535 7th Avenue, SW Olympia, WA 98502 United States |
| 1019 | Dr. Shmuel Shoham | Martin R. Cota; Dr. Daniel L. Herr; Claude William Nogay; Jennifer M. Scarito; Celia M. Taber | Washington Hospital Center 110 Irving Street, NW Washington, DC 20010 United States | Medstar Research Institute Office of Research Integrity Suite 201 6495 New Hampshire Avenue Hyattsville, MD 20783 United States |
| 1021 | Dr. Edgar J. Jimenez | Dr. Philip Anthony Giordano; Dr. Rakesh C. Gupta; Dr. Carlos A. Ruiz; Dr. Orlando Ismael Ruiz Rodriquez; Dr. Jeffrey Adam Sadowsky; Jean S. Schiller; Dr. Antonio Roberto Velardi; Dr. Kurt D. Weber; Dr. Christian C. Zuver | Dr. P. Phillips Hospital  9400 Turkey Lake Road Orlando, FL 32819 United States  Lucerne Pavilion of Orlando Regional Medical Center 818 Main Lane Orlando, FL 32801 United States  Orlando Health 1414 Kuhl Avenue Orlando, FL 32806 United States | IRB of Orlando Health  1414 Kuhl Avenue Orlando, FL 32806 United States |
| 1022 * | Dr. Marc J. Shapiro | Eileen B. Finnin; Debbie A. Fitzgerald; Dr. Michael F. Paccione; Dr. Steven Sandoval | Stony Brook University Medical Center 101 Nicolls Road Stony Brook, NY 11794 United States | Committee on Research Involving Human Subjects W-5530 Melville Library Stony Brook, NY 11794-3368 United States |
| 1023 | Dr. George Marshall Lyon III |  | Emory University Hospital 1364 Clifton Road Atlanta, GA 30322 United States | IRB of Emory University  1599 Clifton Road 5th Floor Atlanta, GA 30322 United States |
| 1024 | Dr. Miren A. Schinco- Schaffer | Dr. Joan L. Huffman; Dr. Andrew J. Kerwin | UF/Shands Jacksonville Medical Center 655 West 8th Street Jacksonville, FL 32209 United States  University of Florida Faculty Clinic 653-2 West 8th Street Jacksonville, FL 32209 United States | Western IRB Inc. 3535 7th Avenue, SW Olympia, WA 98502 United States |
| 1025 * | Dr. Tony N. Hodges | Dr. Ross Bremner; Dr. Michael Smith; Dr. Rajat Walia | Heart and Lung Institute Suite 500 500 W. Thomas Road Phoenix, AZ 85013 United States  St. Joseph's Hospital and Medical Center 350 W. Thomas Road Phoenix, AZ 85013 United States | St. Joseph's Hospital and Medical Center Institutional Review Board for Human Research 350 W Thomas Road Phoenix, AZ 85013 United States |
| 1026 * | Dr. Gyorgy Frendl | Dr. Francis X. Dillon; Dr. Syeda Z. Gardezi; Dr. William B. Gormley; Dr. James P. Hardy; Dr. Peter C. Hou; Dr. Edward Kelly;  Dr. Barrett T. Kitch; Dr. Anthony F. Massaro; Dr. Kimberly A. Matzie; Dr. Shannon Sue McKenna; Dr. Fani Nhuch; Dr. David A. Oxman; Dr. Erika Rangel; Dr. James D. Rawn; Dr. Robert Riviello; Dr. Selwyn O. Rogers; Dr. Nicholas Sandovnikoff; Dr. Naomi Shimizu; Dr. James F. Watkins; Dr. Gerald L. Weinhouse; Dr. Maxwell Weinmann; Dr. Mallory R. Williams; Dr. Joshua H. Winer | Brigham and Women's Hospital 75 Francis Street Boston, MA 02115 United States | Partners Human Research Committee Suite 1002 116 Huntington Avenue Boston, MA 02116 United States |
| 1027 * | Dr. Jennifer K.L. Chow Dr. Susan Hadley (Previous PI) | Dr. Helen Whamond Boucher; Dr. Yoav Golan; Dr. Rakhi Teena Kohli; Dr. Debra Defranca Poutsiaka; Dr. David Richard Snydman | Tufts Medical Center 750 Washington Street Boston, MA 02111 United States | IRB of Tufts Medical Center Box 817 800 Washington Street Boston, MA 02111 United States |
| 1028 | Dr. Nikolaos Almyroudis | Dr. Brahm Hillel Segal | Roswell Park Cancer Institute Elm and Carlton Streets Buffalo, NY 14263 United States | IRB of Roswell Park Cancer Institute Elm and Carlton Streets Buffalo, NY 14263 United States |
| 1029 | Dr. Fernanda de Pinho Silveira Dr. Shahid Husain (Previous PI) | Dr. Karin E. Byers; Dr. Eun Jeong Kwak; Dr. M. Hong Nguyen; Dr. Peter J. Veldkamp; Dr. Emmanuel N. Vergis | University of Pittsburgh Medical Center 200 Lothrop Street Pittsburgh, PA 15213 United States | IRB of University of Pittsburgh Suite 1A 3500 5th Avenue Pittsburgh, PA 15213 United States |
| 1030 | Dr. John Fred Reinhardt | Dr. Alfred Emmanuel Bacon III; Dr. David M. Cohen; Dr. Wesley W. Emmons III; Dr. Stephanie A. Lee; Dr. James S. Ley; Dr. Anand P. Panwalker; Dr. John Paul Piper | Christiana Care Health Care Services 501 West 14th Street Wilmington, DE 19801 United States  Christiana Care Health Services, Christiana Hospital Room 1140 4755 Ogletown Stanton Road Newark, DE 19718 United States  Infectious Disease Associates C-78-80 Omega Drive Newark, DE 19711 United States  Infectious Disease Consultants Apex Medical Center - Suite 201 537 Stanton-Christiana Road Newark, DE 19713 United States | IRB of Christiana Care  MAP 2 - Suite 2114 4735 Ogletown-Stanton Road Newark, DE 19713 United States |
| 1031 | Dr. Sanjay Gopal Revankar | Dr. Jack David Sobel | Detroit Receiving Hospital 4201 St. Antoine Detroit, MI 48201 United States  Harper University Hospital 3990 John R. Street Detroit, MI 48201 United States  University Women's Care Suite 200 3750 Woodward Detroit, MI 48201 United States | Human Investigation Committee 101 East Alexandrine Detroit, MI 48201 United States |
| 1032 * | Dr. Ali Albert El-Solh | Dr. Karin A. Provost; Dr. Sanjay Sethi | Veteran Affairs of Western New York Healthcare System 3495 Bailey Avenue Buffalo, NY 14215 United States | Veterans Affairs Of Western New York Healthcare System Medical Research Building #20 (151) 3495 Bailey Avenue Buffalo, NY 14215 United States |
| 1035 | Dr. Peter Vincente Chin- Hong | Dr. Vicky Dudas; Dr. Richard Alan Jacobs; Dr. Catherine Liu; Dr. Conan Macdougall; Dr. Brian S. Schwartz; Dr. Katherine Y. Yang | UCSF - Mt. Zion Medical Center 1600 Divisadero Street San Francisco, CA 94115 United States  UCSF Department of Medicine Division of Infectious Diseases Room S-410 513 Parnassus Avenue San Francisco, CA 94143 United States  UCSF Medical Center 505 Parnassus Avenue San Francisco, CA 94143 United States  UCSF Medical Center 500 Parnassus Avenue San Francisco, CA 94143 United States | UCSF Committee on Human Research Office of Research Suite 315 - Box 0962 3333 California Street San Francisco, CA 94118 United States |
| 1036 * | Dr. Kieren Anne Marr (Previous PI) Dr. Lynne M. Strasfeld |  | Oregon Health & Sciences University 3181 SW Sam Jackson Park Road Portland, OR 97239 United States | IRB of Oregon Health & Sciences University 2525 Southwest 1st Avenue Portland, OR 97239 United States |
| 1037 | Dr. Felicia Aurelia Ivascu | Dr. Paul Guy Bove; Dr. Matthew D. Sims | William Beaumont Hospital Surgical Clinical Trials Office 3811 West 13 Mile Road Royal Oak, MI 48073 United States  William Beaumont Hospital 3601 West 13 Mile Road Royal Oak, MI 48073 United States | William Beaumont Hospital Human Investigation Committee 3811 West 13 Mile Road Royal Oak, MI 48073 United States |
| 1039 * | Dr. Tawanda Gumbo | Dr. Kathryn Dzintars; Amit D. Gode; Dr. Mark A. Swancutt | Parkland Health and Hospital System 5201 Harry Hines Boulevard Dallas, TX 75235 United States  University of Texas Southwestern Medical Center at Dallas 5323 Harry Hines Boulevar Dallas, TX 75390 United States | IRB of University of Texas Southwestern Medical Center at  Dallas 5323 Harry Hines Boulevard Dallas, TX 75390-8843 United States |
| 1040 | Dr. Victor Eduardo Mulanovich | Dr. Javier A. Adachi; Dr. Bruno P. Granwehr; Dr. Ray Y. Hachem; Dr. Dimitrios P. Kontoyiannis; Dr. Issam Inaam Raad | The University of Texas MD Anderson Cancer Center 1515 Holcombe Boulevard Houston, TX 77030 United States | The University Of Texas MD Anderson Cancer Center Office of Protocol Research Unit 1009 1515 Holcombe Boulevard Houston, TX 77030 United States |
| 1041 | Dr. Jo-Anne Hertha Young |  | University of Minnesota Medical Center, Fairview 420 Delaware Street SE Minnesota, MN 55455 United States | University of Minnesota Research Subjects' Protection Program Mayo Mail Code 820 420 Delaware Street SE Minneapolis, MN 55455 United States |
| 1042 * | Dr. Thomas Frost Patterson | Dr. Gregory Michael Anstead; Deborah K. Berg; Dr. Delia Elizabeth Bullock; Dr. Jose A. Cadena; Dr. Anthony W. Hartzler; Dr. James S. Lewis; Dr. George R. Thompson | UHC-DT - University Health Center Downtown 527 North Leona Street San Antonio, TX 78284 United States  University Hospital 4502 Medical Drive San Antonio, TX 78229 United States  University of Texas Health Science Center Medicine ID MC-7881 7703 Floyd Curl Drive San Antonio, TX 78229 United States  UT Medicine San Antonio Diagnostic Pavillion 4627 Medical Drive San Antonio, TX 78229 United States | IRB at The University of Texas Health Science Center at San Antonio 7703 Floyd Curl Drive San Antonio, TX 78229 United States |
| 1043 | Dr. Elias Jamal Anaissie | Dr. Syed Abbas Ali; Dr. Yazan Alsayed; Dr. Bart N. Barlogie; Dr. Divaya Bhutani; Dr. Monica L. Grazziutti; Dr. Klaus Hollmig; Dr. Ali Javed; Dr. Mehmet Kocoglu; Dr. Pooja Motwani; Dr. Bijay P. Nair; Dr. Mauricio Pineda- Roman; Dr. Frits Van Rhee; Dr. Sarah Waheed | Myeloma Institute for Research and Therapy University of Arkansas for Medical Sciences 4301 West Markham Little Rock, AR 72205 United States | IRB of University of Arkansas for Medical Sciences  University of Arkansas for Medical Sciences Suite 636 4301 West Markham Street Little Rock, AR 72205-7199 United States |
| 1045 * | Dr. Suneel Khetarpal | Shacreyett V. Burton; Dr. David J. Ciesla; Denise A. Fife; Natalie Giarelli; Ann M. Hintz;Dr. Luis E. Llerena | Center for Outpatient Research Excellence Suite 470 5 Tampa General Circle Tampa, FL 33606  United States  Tampa General Hospital 1 Tampa General Circle Tampa, FL 33606  United States | Western IRB Inc. 3535 7th Ave, SW  Olympia, WA 98502-5010 United States |
| 1048 * | Dr. Ronald Glenn Washburn | Dr. Laurie Robin Grier; Dr. John William King; Dr. Robert L. Penn; Dr. John Rucker Todd; Dr. Ulysses S. Wu | Louisiana State University Health Sciences Center – Shreveport 1501 Kings Highway Shreveport, LA 71103 United States | IRB #2 of Louisiana State University Health Sciences Center-Shreveport Building 2 2627 Linwood Avenue Shreveport, LA 71103  United States |
| 1049 | Dr. Donald Richardson Graham | Holly E. Heffren; Dr. Douglas Ray Leigh; Dr. Praveen K. Mullangi; Dr. Steven Douglas O'Marro | Memorial Medical Center 701 North 1st Street Springfield, IL 62702 United States  Springfield Clinic 1025 South 6th Street Springfield, IL 62703 United States  Springfield Clinic Infectious  Diseases St. John's Pavilion, 1st Floor 301 North 8th Street Springfield, IL 62701 United States  Springfield Clinic Research Department Main Campus West Building - Suite 4300 1025 South 6th Street Springfield, IL 62703 United States  St. John's Hospital 800 East Carpenter Street Springfield, IL 62702 United States | IRB of Fox Suite 202 133 South 4th Street Springfield, IL 62701 United States |
| 1050 * | Dr. Simon J. Tsiouris | Steven J.I. Chang; Dr. Christine J. Kubin; Dr. Magdalena E. Sobieszczyk; Dr. Michael T. Yin | Columbia University Medical Center Research Pharmacy - BB Satellite William Black Building 650 West 168th Street Basement B-30 New York, NY 10032 United States  New York Presbyterian Hospital - Columbia University Medical Center Milstein Hospital Building 177 Fort Washington Avenue New York, NY 10032 United States  New York Presbyterian Hospital - Columbia University Medical Center Herbert Irving Pavilion 161 Fort Washington Avenue New York, NY 10032 United States | IRB of Columbia University Medical Center  4th Floor 722 West 168th Street New York, NY 10032 United States  Western IRB Inc. 3535 7th Avenue, SW Olympia, WA 98502-5010 United States |
| 1051 * | Dr. Luis Ostrosky- Zeichner | Dr. Sujatha Krishnan; Sibyl Ward-Stanberry | Memorial Hermann Hospital 6411 Fannin Street Houston, TX 77030 United States | The University of Texas Health Science Center at Houston Office of Research Support Committees Suite 1100 6410 Fannin Houston, TX 77030 United States |
| 1056 | Dr. Aruna Kumar Subramanian | Dr. Paul Gisbert Auwaerter; Dr. Kieren Anne Marr; Dr. Michael Melia; Dr. Dionissios Neofytos;  Dr. Mark S. Sulkowski | Johns Hopkins Hospital 600 North Wolfe Street Baltimore, MD 21287  United States  Johns Hopkins Medicine Moore Clinic Carnegie 346 600 North Wolfe Street Baltimore, MD 21287 United States  Johns Hopkins University Clinical Trials Unit Room 205 and 107 550 North Broadway Baltimore, MD 21205 United States  Johns Hopkins University Investigational Drug Pharmacy 600 North Wolfe Street Osler 100 Baltimore, MD 21205 United States | IRB of Johns Hopkins Office of Human Subjects Research Reed Hall  Suite B-130 1620 McElderry Street Baltimore, MD 21205-1911 United States |
| 1058 | Dr. Kent James Stock | Dr. Ludwig Alexander Lettau; Dr. Randall D. Marosok | Low Country Infectious Diseases 1938 Charlie Hall Boulevard Charleston, SC 29414 United States  Roper Hospital 316 Calhoun Street Charleston, SC 29401 United States | IRB of Roper St. Francis Hospital 316 Calhoun Street Charleston, SC 29401  United States |
| 1059 * | Dr. Samuel A. Lee | Dr. Marcos A. Burgos; Dr. Diane E. Goade; Dr. Michelle J. Iandiorio; Dr. Tsun Sheng Neil Ku; Dr. Gregory James Mertz | University of New Mexico Clinical Trials Center SURGE Rm 140, MSC08 4560 2701 Frontier Place, NE  Albuquerque, NM 87131 United States  University of New Mexico Hospital 211 Lomas Boulevard Albuquerque, NM 87106 United States | University of New Mexico Human Research Review  Committee  MSCO8 4560 BMSB Room B-79 One University of New Mexico Albuquerque, NM 87131-0001 United States |

* Did not randomize patients
